# Class I Major Histocompatibility Complex, the Trojan Horse for Secretion of Amyloidogenic β_2_-Microglobulin*

**DOI:** 10.1074/jbc.M113.524157

**Published:** 2013-12-13

**Authors:** Levon Halabelian, Stefano Ricagno, Sofia Giorgetti, Carlo Santambrogio, Alberto Barbiroli, Sara Pellegrino, Adnane Achour, Rita Grandori, Loredana Marchese, Sara Raimondi, P. Patrizia Mangione, Gennaro Esposito, Raya Al-Shawi, J. Paul Simons, Ivana Speck, Monica Stoppini, Martino Bolognesi, Vittorio Bellotti

**Affiliations:** From the ‡ Dipartimento di Bioscienze, Università di Milano, Via Celoria 26, 20133 Milano, Italy,; the § Department of Molecular Medicine, Institute of Biochemistry “A. Castellani”, Via Taramelli 3/b, 27100 Pavia, Italy,; the ¶ Department of Biotechnology and Biosciences, University of Milano-Bicocca, Piazza della Scienza 2, 20126 Milan, Italy,; the ‖ Section of Biochemistry, Dipartimento di Scienze Molecolari Agroalimentari, University of Milan, 20133 Milan, Italy,; the ** Dipartimento di Scienze Farmaceutiche- sez. Chimica generale e organica “A. Marchesini”, Università degli Studi di Milano, 20133 Milan, Italy,; the ‡‡ Center for Infectious Medicine (CIM), Department of Medicine, Karolinska University Hospital Huddinge, Karolinska Institutet, 141 86 Stockholm, Sweden,; the §§ Wolfson Drug Discovery Unit, Centre for Amyloidosis and Acute Phase Proteins, University College London, London NW3 2PF, United Kingdom,; the ¶¶ Dipartimento di Scienze Mediche e Biologiche, Università di Udine, 33100 Udine, Italy, and; the ‖‖ Centre for Biomedical Science, Division of Medicine, University College London, London NW3 2PF, United Kingdom

**Keywords:** Amyloid, Major Histocompatibility Complex (MHC), Protein Aggregation, Protein Complexes, Protein Structure, Beta2 Microglobulin Amyloidosis, D76N Beta2-Microglobulin Variant

## Abstract

To form extracellular aggregates, amyloidogenic proteins bypass the intracellular quality control, which normally targets unfolded/aggregated polypeptides. Human D76N β_2_-microglobulin (β_2_m) variant is the prototype of unstable and amyloidogenic protein that forms abundant extracellular fibrillar deposits. Here we focus on the role of the class I major histocompatibility complex (MHCI) in the intracellular stabilization of D76N β_2_m. Using biophysical and structural approaches, we show that the MHCI containing D76N β_2_m (MHCI_76_) displays stability, dissociation patterns, and crystal structure comparable with those of the MHCI with wild type β_2_m. Conversely, limited proteolysis experiments show a reduced protease susceptibility for D76N β_2_m within the MHCI_76_ as compared with the free variant, suggesting that the MHCI has a chaperone-like activity in preventing D76N β_2_m degradation within the cell. Accordingly, D76N β_2_m is normally assembled in the MHCI and circulates as free plasma species in a transgenic mouse model.

## Teaser

**Background:** Amyloidogenic D76N β_2_m variant escapes the intracellular quality control despite its instability.

**Results:** We show tridimensional structure and stability of D76N β_2_m assembled within MHCI compared with the wild type protein.

**Conclusion:** Assembly of D76N β_2_m within the MHCI totally masks its misfolding propensity.

**Significance:** The MHCI-mediated stabilization of amyloidogenic D76N β_2_m explains the failure of quality control in preventing its secretion.

## Introduction

Amyloid-related diseases comprise an ever growing class of human pathologies in which fold impairment or unfolding of a specific protein lead to extracellular protein aggregation (1). To protect from intracellular protein misfolding, human cells adopt sophisticated recovery systems, either to help protein molecules fold correctly or to remove and destroy unfolded/incorrectly folded polypeptides with specific proteolytic pathways (2). Although the number of proteins causing misfolding diseases in humans is a small subset of the whole proteome, amyloidogenic proteins escape the quality control machinery. In some cases proteins become amyloidogenic due to post-translational modifications, which may occur after they have passed the cellular quality checks. For example, the amyloid β peptide, associated with Alzheimer disease, is generated by subcellular proteolytic processing of the β-amyloid precursor protein within the well known amyloidogenic pathway and then secreted (3). On the other hand, amyloidogenic proteins often fold properly and turn into pathologic aggregates slowly, over many years (4). A typical example of the latter is wild type β_2_-microglobulin (WT β_2_m),^4^ which is the etiological agent of dialysis-related amyloidosis (5).

WT β_2_m is a well folded 99-residue protein, adopting a β-sandwich immunoglobulin fold, which is very stable under physiological conditions. β_2_m is the light chain of the class I major histocompatibility complex, a stable ternary complex that also comprises the heavy chain and an 8–11-residue peptide bound by the heavy chain (6). The MHCI is assembled in the endoplasmic reticulum and thereafter transported to the extracellular side of the cell membrane, to which MHCI is anchored by a short transmembrane domain (7). During its normal turnover, the MHCI continuously releases its β_2_m subunits, which are ultimately cleared only via the kidney.

Amyloid aggregation by WT β_2_m occurs in patients with end stage renal failure undergoing chronic hemodialysis treatment in whom the protein circulates at persistently raised concentrations (up to 30–40-fold the normal levels) and accumulates in fibrillar aggregates in bones and joints (5). Recently, a new form of fatal hereditary systemic amyloidosis, caused by previously unknown D76N β_2_m mutation, was discovered (8). The presentation of this severe disease is quite different from dialysis-related amyloidosis; patients heterozygous for the D76N β_2_m mutation suffer from a systemic disease involving all tissues, except the central nervous system and the skeleton (8). The D76N mutation remarkably decreases the stability of the variant protein and dramatically increases β_2_m aggregation propensity under physiological conditions (8).

The endoplasmic reticulum is known to host a complex homeostatic system referred to as the unfolded protein response (UPR), which targets unfolded/aggregated protein molecules to degradation by the ubiquitin-proteasome pathway (2). Consequently, a protein as unstable and prone to aggregation as the D76N β_2_m variant should trigger the UPR system and be efficiently degraded. However, the presence of the variant in the plasma and the large amyloid deposits found in the extracellular space of almost all the tissues are consistent with efficient secretion of the D76N variant from the cells expressing MHCI (8).

To gain insight into the mechanisms that allow D76N β_2_m molecules to escape the UPR clearance system and to characterize the effects of the D76N mutated light chain on the assembled MHCI (hereinafter named MHCI_76_), a thorough structural and biophysical study on a human MHCI_76_ was undertaken. The stability of MHCI_76_ as compared with the WT MHCI has been assessed by circular dichroism and mass spectrometry. Also the crystal structure of the MHCI_76_, in association with a nonapeptide, has been determined at 2.65 Å resolution and compared with the crystal structure of WT MHCI (bearing WT β_2_m and the same peptide), here reported at 3.1 Å resolution.

Furthermore, the dynamics of both WT and D76N β_2_m, either as free species or as assembled MHCI, have been studied by limited proteolysis. Although the experiments on monomeric species confirm the remarkable differences in stability and dynamics between WT and variant β_2_m (9), the interactions within the MHCI complex evidently control the conformational variability and dynamics of the variant. Furthermore, we used a transgenic mouse model expressing the D76N β_2_m variant to demonstrate that the intracellular assembly of the MHCI, as well as its translocation to the membrane, is not affected by the instability or the misfolding propensity of the D76N β_2_m variant.

## EXPERIMENTAL PROCEDURES

### 

#### 

##### Peptide Synthesis

The two nonapeptides (peptide I YLLMWITQV; peptide II SLYAEDTAV) were prepared by microwave-assisted solid phase synthesis (10) based on Fmoc chemistry on preloaded 2-chlorotrityl resin (1.5 meq/g substitution) using a 5-fold molar excess of 0.2 m Fmoc-protected amino acids dissolved in dimethylformamide and using *N*-hydroxybenzotriazole/*O*-(benzotriazol-1-yl)-*N*,*N*,*N*′,*N*′-tetramethyluronium hexafluorophosphate/diisopropylethylamine (5:5:10) as activators. Coupling reactions were performed for 5 min at 40 watt with a maximum temperature of 75 °C. Deprotection was performed in two stages using 20% piperidine in dimethylformamide (5 and 10 min each). Cleavage was performed using 10 ml of reagent K (TFA/phenol/water/thioanisole/ethanedithiol; 82.5/5/5/5/2.5) for 180 min. Following cleavage, peptides were precipitated out and washed using ice-cold anhydrous ethyl ether. All peptides were purified by reverse phase HPLC using a gradient elution of 5–70% solvent B (solvent A: water/acetonitrile/TFA 95/5/0.1; solvent B: water/acetonitrile/TFA 5/95/0.1) over 20 min at a flow rate of 10 ml/min. The purified peptides were freeze-dried and stored at 0 °C.

##### MHCI Purification

The HLA-A0201 heavy chain and the two β_2_m variants were expressed separately as inclusion bodies using the BL21 (DE3) *Escherichia coli* strain, as described previously (11). Inclusion bodies were solubilized in 6 m guanidinium hydrochloride, 20 mm Tris-HCl, pH 8.0, and purified by size exclusion chromatography using a Superdex 75 column. The MHCI component subunits were refolded together by adding 2 μm β_2_m, 1 μm HLA-A0201 heavy chain, and 10 μm peptide into the refolding buffer (100 mm Tris-HCl, 480 mm l-arginine HCl, 2 mm EDTA, 0.5 mm oxidized glutathione, 5 mm reduced glutathione, 0.5 mm 4-(2-aminoethyl)-benzenesulfonyl fluoride hydrochloride, pH 8.0) and incubated on a magnetic stirrer at 4 °C for 48 h. The refolded MHCI solution was concentrated by tangential filtration system and purified by size exclusion chromatography using a Superdex 200 column and elution buffer 150 mm NaCl, 20 mm Tris-HCl, pH 8.0. The eluted fractions were analyzed by static light scattering and SDS-PAGE. The presence of D76N β_2_m was confirmed by chymotrypsin digestion and peptide sequencing by mass spectrometry (12).

##### Circular Dichroism

Thermal stability experiments were carried out by circular dichroism (CD) on a J-810 spectropolarimeter (JASCO Corp., Tokyo, Japan) equipped with a Peltier system for temperature control. All measurements on MHCI samples were performed in 150 mm sodium chloride, 20 mm Tris-HCl, pH 8.0, at 0.2 mg/ml protein concentration. The temperature ramp measurements were recorded from 20 to 95 °C (temperature slope 1.0 °C/min) in a 0.1-cm path length cuvette and monitored at 218-nm wavelength. Monomeric D76N β_2_m was studied under the same conditions previously used for monomeric WT β_2_m (13).

##### Mass Spectrometry

Nano-ESI-MS experiments were performed on a hybrid quadrupole time-of-flight mass spectrometer (QSTAR Elite, AB-Sciex, Foster City, CA) equipped with a nano-electrospray ionization source. Samples were infused at 10 μm protein concentration in 100 mm ammonium acetate, using metal-coated borosilicate capillaries with emitter tips of 1-μm internal diameter (Proxeon, Odense, Denmark). The pH of the solution was adjusted by the addition of formic acid to the indicated values. The following instrumental setting was applied: declustering potential 50–110 V, ion spray voltage 1000–1200 V, and curtain gas pressure 20 psi. The sample source and the instrument interface were kept at room temperature.

##### Crystallization and Structure Determination

Crystals of MHCI_76_ and WT MHCI, both bearing the NY-ESO-1-derived epitope pI (YLLMWITQV), were grown at 20 °C with sitting drop techniques by mixing equal amounts of a 5–7 mg/ml protein solution, and the reservoir solution (1.9 m ammonium sulfate, 2% PEG 6000, 100 mm Tris-HCl, pH 8.0–8.2). Crystals were cryoprotected with 20–33% glycerol and flash-frozen in liquid nitrogen. X-ray diffraction data were collected at the beam lines ID14-4 and BM-14 (European Synchrotron Radiation Facility (ESRF), Grenoble, France) for MHCI_76_ and WT MHCI, respectively. X-ray data were processed using MOSFLM and SCALA (14, 15). The structure of MHCI_76_ was solved by molecular replacement using PHASER (16); the crystal structure of human MHCI by Webb *et al.* (17) (Protein Data Bank (PDB) code 1S9W) was chosen as the search model after removal of the MHCI bound peptide.

To determine the WT MHCI structure, whose crystals are isomorphous with those of MHCI_76_, a difference Fourier analysis based on phases calculated from the MHCI_76_ structure was first carried out. Using the Xtriage program (18), a pseudo-merohedral twinning was detected for both MHCI crystals (MHCI_76_ twinning fraction = 18.5%, WT MHCI twinning fraction = 31.6%). The structures were refined using Phenix.refine (18) and REFMAC5, applying the twin refinement protocol (19).

TLS group refinement and Non-Crystallographic Symmetry (NCS) restrains were introduced throughout the refinement. Model building and analysis of the WT MHCI and MHCI_76_ structures were carried out using COOT (Crystallographic Object-Oriented Toolkit) (20); figures were prepared with the CCP4MG software (21). Structure factors and coordinates have been deposited in the Protein Data Bank under accession codes 4L3C for MHCI_76_ pI and 4L29 for WT MHCI pI.

##### Limited Proteolysis

Comparative limited proteolysis experiments were performed with monomeric WT β_2_m and D76N β_2_m and on the assembled WT MHCI and MHCI_76_. Experiments were carried out by incubating the samples with trypsin (Sigma-Aldrich) in 50 mm ammonium bicarbonate (pH 7.5) at 37 °C, using enzyme-to-substrate ratios ranging between 1:20 and 1:500 (w/w).

The extent of the reaction was monitored on a time course basis by sampling the incubation mixture at different time intervals. Peptide mixtures were analyzed by MALDI-TOF mass spectrometry using a Micromass spectrometer (Waters) in linear mode. The sample was solubilized in 0.2% trifluoroacetic acid, and the protein solution was mixed 1:1 with a solution of α-cyano-4-hydroxycinammic acid, 5 mg/ml in acetonitrile, 0.2% TFA 7:3 (v/v), applied onto the metallic sample plate, and air-dried. Mass calibration was performed using a sample of recombinant WT β_2_m as standard.

##### Transgenic Mice Expressing Human β_2_m

A wild type copy of the human β*_2_m* gene was amplified from genomic DNA using Phusion DNA polymerase, cloned, and sequence-verified. The D76N mutation was then introduced by site-directed mutagenesis (22).

Following sequence verification, transgenic mice were generated by pronuclear microinjection of C57BL/6J embryos with the mutated β*_2_m* gene, including 2.1 kb and 630 bp of 5′- and 3′-flanking sequences, respectively. Transgenic mice were identified by PCR using human β_2_m-specific primers ACTGAATTCACCCCCACTGA and ATGGGATGGGACTCATTCAG. Five independent transgenic mice were obtained, and lines were established from four of them.

##### Effect of Trypsin on D76N β_2_m Assembled in Natural MHCI

Leukocytes were gently extracted from the spleen of two transgenic mice expressing D76N β_2_m and separated by Ficoll density gradient centrifugation. A suspension containing ∼240 × 10^−6^ cells/ml was analyzed by Western blot following 15% PAGE carried out under denaturing and reducing conditions, and the band corresponding to monomeric human β_2_m was quantified by densitometry. Based on the resulting concentration of 4 μg/ml, the cell suspension was exposed to trypsin at 1:20 w/w enzyme:substrate ratio alongside a solution of monomeric D76N β_2_m in the same experimental conditions. Aliquots at time 0, 30, 60, 120, and 240 min were analyzed by Western blot developed with a rabbit polyclonal anti-human β_2_m (2.4 μg/ml, Dako) and polyclonal anti-rabbit IgG peroxidase conjugate (2.5 × 10^−5^ μg/ml, Dako) as primary and secondary antibody, respectively, and detected with chemiluminescent substrate (Western Immobilon, Millipore).

## RESULTS

### 

#### 

##### MHCI Reconstitution

To explore potential effects on MHCI stability by D76N variant β_2_m, as compared with the WT species, the prototypic heavy chain of MHCI, HLA-A0201 (HLA-A2), was refolded in the presence of each of the two β_2_m variants and of two different peptides. The selected epitopes NY-ESO-1(Y^1^V^9^) and FR-20(Y^3^) are modified peptide ligand variants from the melanoma-associated antigen NY-ESO-1 (23) and multiple myeloma-associated antigen FR-20 (24), respectively. Both epitopes have been modified to enhance the overall stability of HLA-A0201 complexes. Although peptide positions 1 and 9 were mutated to Tyr and Val (phospho-Tyr^1^ and phospho-V^9^, respectively) in NY-ESO-1(Y^1^V^9^), residue 3 was changed to Tyr (phospho-Tyr^3^) in FR-20. The four complexes thus produced were refolded using adapted well established protocols (11), resulting in efficient production of the HLA-A2/WT β_2_m/NY-ESO-1(Y^1^V^9^), HLA-A2/D76N β_2_m/NY-ESO-1(Y^1^V^9^), HLA-A2/WT β_2_m/FR-20(Y^3^), and HLA-A2/D76N β_2_m/FR-20(Y^3^) complexes, hereafter named WT MHCI pI, MHCI_76_ pI, WT MHCI pII, and MHCI_76_ pII, respectively.

##### Overall Thermodynamic Stability by Circular Dichroism

**FIGURE 1.**
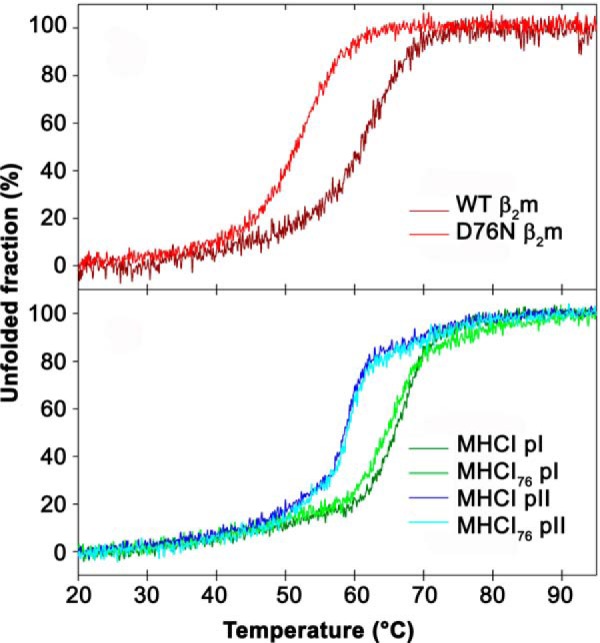
**Thermodynamic stability by circular dichroism.** *A* and *B*, variation of far-UV CD signal as a function of temperature. *A*, thermal unfolding of monomeric WT and D76N β_2_m monitored at 202 nm. *B*, thermal unfolding of WT MHCI and MHCI_76_ with either pI or pII monitored at 218 nm. Melting temperatures for monomeric proteins and their assemblies as assessed using CD curves are reported in Table 1.

To assess the possible effects of D76N β_2_m variant in the context of MHCI stability, temperature ramps for WT MHCI and MHCI_76_, both in complex with either pI or pII, were performed by monitoring protein unfolding by ellipticity in the far UV region. The two different peptides were employed to acquire a general trend in WT MHCI *versus* MHCI_76_ stability and to rule out specific effects due to the chosen peptide sequence. Fig. 1*A* shows the thermal unfolding of the isolated monomeric WT β_2_m and of the D76N variant; the resulting sigmoid curves clearly highlight different thermal stabilities for the two variants (see also Table 1 for *T_m_* values). The thermal unfolding curves for WT MHCI and MHCI_76_, presenting the same peptide, are almost perfectly superimposable; conversely, both WT MHCI and MHCI_76_ sigmoid curves are markedly shifted according to the nature of the peptide bound to the heavy chain (Fig. 1*B*). Thus, under these experimental conditions, the D76N mutation does not affect the overall thermal stability of the assembled MHCI complexes (Fig. 1 and Table 1).

**TABLE 1:** **Melting temperatures** *T_m_* values are assessed on far UV CD curves (Fig. 1).

	Monomer	MHCI pI	MHCI pII
WT β_2_m	62.4 °C*^a^*	67.3 °C	58.8 °C
D76N β_2_m	52.8 °C	65.5 °C	59.1 °C

*^a^* As in Santambrogio *et al.* (13).

##### Relative Stability by Mass Spectrometry

**FIGURE 2.**
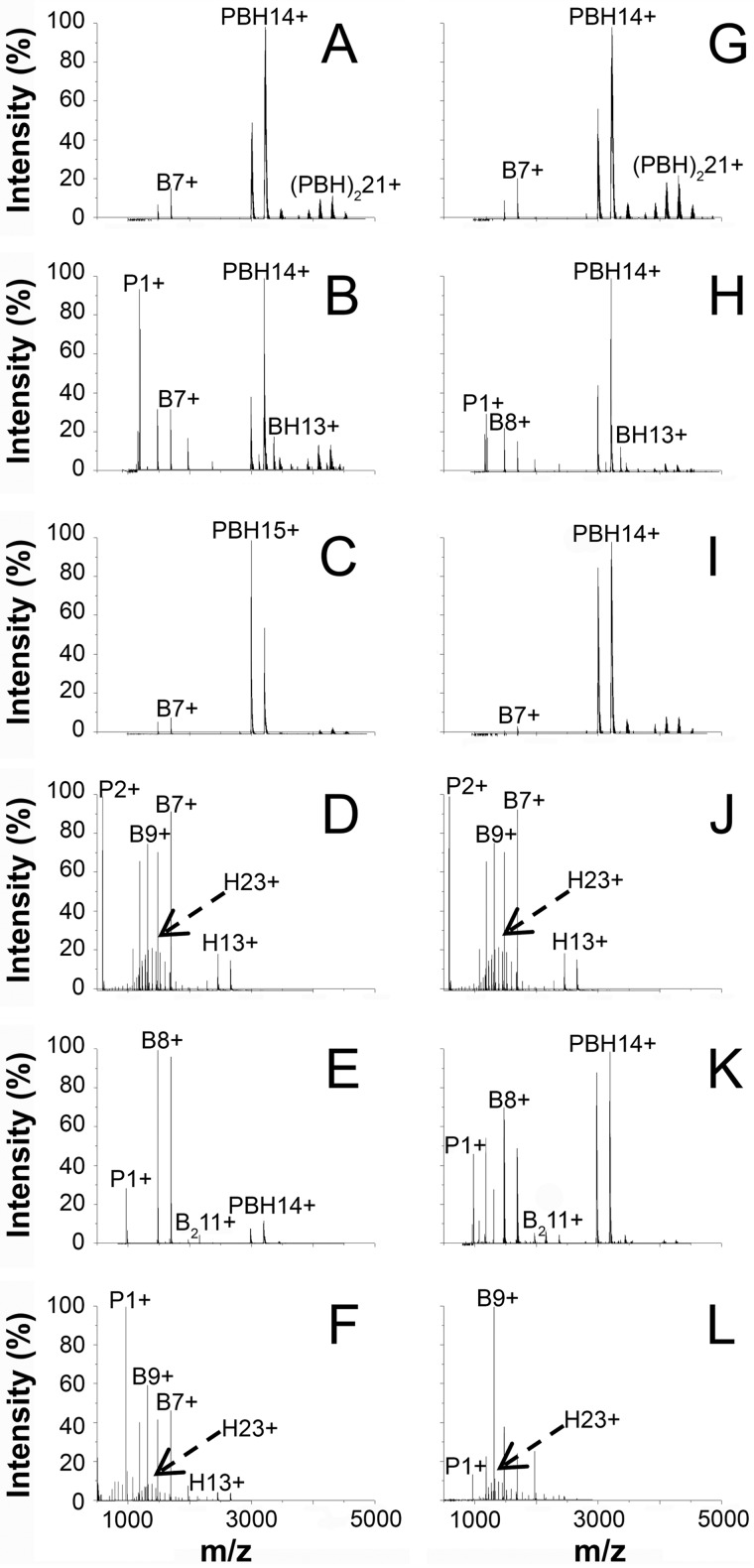
**Relative stability by mass spectrometry.** *A–L*, nano-ESI-MS spectra of WT MHCI (*A–F*) or MHCI_76_ (*G–L*) in 100 mm ammonium acetate. *A* and *G*, pH 7, DP 50 V, peptide I. *B* and *H*, pH 7, DP 110 V, peptide I. *C* and *I*, pH 5, DP 50 V, peptide I. *D* and *J*, pH 3.5, DP 50 V, peptide I. *E* and *K*, pH 5, DP 50 V, peptide II. *F* and *L*, pH 3.5, DP 50 V, peptide II. Main peaks are labeled with the molecular species and net charge. *P*, peptide; *B*, β_2_m; *H*, heavy chain; *PBH*, entire MHCI complex.

All the refolded complexes were analyzed by nano-ESI-MS under nondenaturating or under denaturing conditions, to analyze stoichiometry, conformational states, relative stabilities, and dissociation patterns (25–28). The spectra of WT MHCI and MHCI_76_ samples are very similar (Fig. 2). Under nondenaturing conditions (Fig. 2, *A* and *G*), the predominant signals are those of the ternary complexes, containing β_2_m, the heavy chain, and the peptide. The measured mass is ∼45,140 Da for WT MHCI pI and ∼45,121 Da for MHCI_76_ pI, slightly higher than their respective calculated masses (44,927 and 44,926 Da). Such a discrepancy is typically observed for noncovalent complexes detected by mass spectrometry, and can be explained by the trapping of solvent molecules at protein interfaces (29). The charge-state distribution is narrow and unimodal, centered on the 14^+^ ion in each case (Fig. 2, *A* and *G*). Such features indicate that WT MHCI pI and MHCI_76_ pI are folded in compact, native conformations. The ternary complex itself appears to be prone to self-association, giving rise to peak envelopes corresponding to MHCI dimers. Furthermore, signals of free and folded β_2_m are present in the *m*/*z* range of 1000–2000 (13). Because this is the only monomeric component detectable under the employed experimental conditions, it is likely due to the presence of some free β_2_m in the original sample, rather than to dissociation during electrospray. These results indicate that the D76N mutation does not induce major changes in the MHCI structure and assembly.

The complexes can be dissociated by increasing the declustering potential (DP) at the instrument interface, resulting in higher internal energy of the desolvated ions. The two MHCI variants respond in a similar way. Fig. 2, *B* and *H*, show representative spectra at intermediate DP values (110 V), at which new signals can be recognized, indicating accumulation of free peptide and binary β_2_m/heavy chain complexes. No signals are detectable corresponding to the peptide associated with either one of the two protein subunits, indicating that the complex dissociation occurs preferentially with initial loss of the peptide, regardless of the β_2_m variant. Thus, both WT MHCI and MHCI_76_ appear to be characterized by a similar internal hierarchy and similar gas phase stability. The two pI complexes also display similar stabilities in solution as a function of pH, being still preserved at pH 5 (Fig. 2, *C* and *I*) and completely dissociated into their individual components at pH 3.5 (Fig. 2, *D* and *J*). The measured, isotope-averaged molecular masses are 1166 Da for peptide I, 11,860 Da for WT β_2_m, 11,859 Da for D76N β_2_m, and 31,901 Da for the heavy chain, matching the respective calculated values to an accuracy below 75 ppm. As shown in Fig. 2, *D* and *J*, the free proteins display bimodal charge-state distributions at pH 3.5, indicating the existence of large amounts of the denatured forms.

The analysis described above was extended to WT MHCI pII and MHCI_76_ pII complexes, which were analyzed under several different solvent and instrument conditions. Again, the response was not significantly affected by the β_2_m variant present in the complex. However, major differences were detected depending on the presence of pII, particularly in response to pH titrations.

Spectra at pH 5 (Fig. 2, *E* and *K*) show that the pII complexes, in contrast to the ones with pI, already dissociate into the single components. The measured mass of WT MHCI in this case is ∼44,820 Da (calculated mass 44,729 Da), and that of MHCI_76_ is ∼44,817 Da (calculated mass 44,728 Da). Dissociation is complete at pH 3.5 (Fig. 2, *F* and *L*), for both WT MHCI pII and MHCI_76_ pII. Overall, these results indicate that the presence of different peptides affects the relative stability of the complexes, whereas no obvious effects of the D76N mutation on MHCI stability or internal hierarchy of the complex could be detected under all the conditions tested. These findings are entirely consistent with the relative stabilities of the different complexes measured by circular dichroism reported above.

##### Comparative Analysis of MHCI_76_ pI and WT MHCI pI Crystal Structures

**FIGURE 3.**
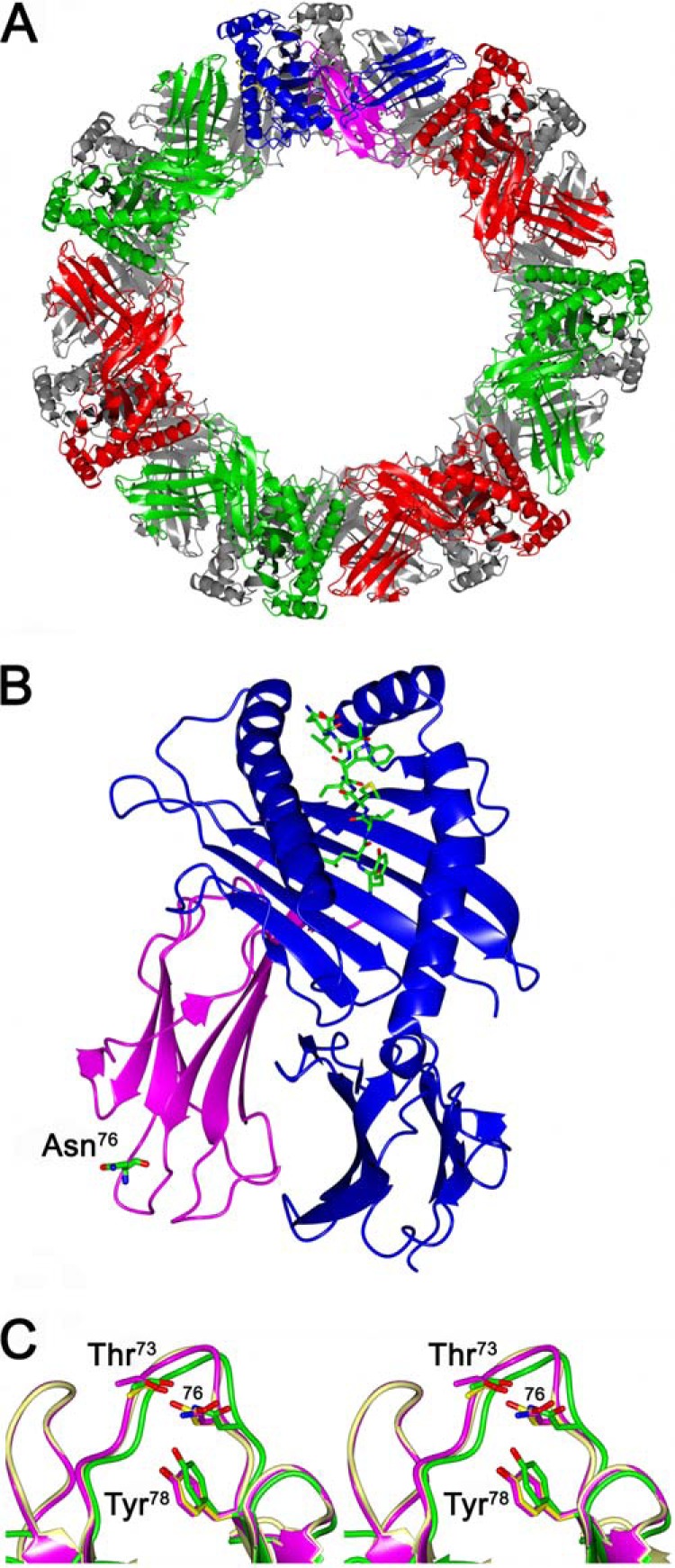
**Comparative analysis of MHCI_76_ pI and WT MHCI pI crystal structures.** *A*, ribbon representation of the MHCI_76_ pI assembly in the asymmetric unit. The 14 MHCI molecules are organized in two juxtaposed heptameric rings. MHCI units from the front ring are differently colored; those in the back are *gray. B*, ribbon representation of one MHCI_76_ unit structure. The D76N mutation site on β_2_m is detailed as a sticks model. The heavy chain, light chain, and bound nonapeptide are in *blue*, *magenta*, and *green*, respectively. *C*, stereo representation of the β_2_m EF loop from the MHCI_76_ structure (*magenta*), from WT MHCI (*light grey*), and from the monomeric D76N β_2_m (*green*).

Crystal structures of MHCI_76_ pI and of WT MHCI pI were determined at 2.65 and 3.1 Å resolution, respectively. MHCI_76_ pI and WT MHCI pI produced isomorphous crystals belonging to the orthorhombic space group P2_1_2_1_2_1_. The asymmetric unit contains 14 MHCI moieties, arranged in two juxtaposed heptameric rings (Fig. 3*A*), a previously unreported packing for MHCI crystal structures, containing 69% solvent content. The assembled MHCI_76_ 14-mer, one MHCI complex, and the mutated Asn^76^ residue region in the protein are shown in Fig. 3.

In both crystal structures, the electron density is of excellent quality for all the 14 MHCI units, except for the 87–91-residue stretch in all 14 HLA-A2 molecules. Data collection and refinement statistics for MHCI_76_ pI and WT MHCI pI are shown in Table 2. The 14 MHCI_76_ pI molecules in the asymmetric unit show negligible structural differences, and the average root mean square deviation was 0.29 Å/374 C^α^ atoms between MHCI complexes.

**TABLE 2:** **Data collection and refinement statistics for WT MHCI and MHCI_76_** Values in parenthesis are for the highest resolution shell.

	Structure (PDB entry)	
	WT MHCI (4L29)	MHCI_76_ (4L3C)
**Data collection**		
Beam line	BM-14 (ESRF)	ID14–4 (ESRF)
Space group	Orthorhombic P2_1_2_1_2_1_	Orthorhombic P2_1_2_1_2_1_
Unit cell constants (Å)	*a* = 101.5 *b* = 313.3 *c* = 314.4	*a* = 102.1 *b* = 314.5 *c* = 316.2
Solvent content (%)	69.0	69.5
Resolution (Å)	53.88–3.10 (3.27–3.10)	70.63–2.65 (2.79–2.65)
*R*_merge_*^a^* (%)	19.2 (101.5)	16.8 (90.5)
I/sig(I)	9.3 (2.2)	6.8 (1.9)
Completeness (%)	100.0 (100.0)	99.7 (99.7)
Redundancy	5.8 (5.5)	5.3 (5.4)
Unique reflections	182,537 (26405)	293,400 (42513)

**Refinement**		
*R*_work_*^b^* (%)	17.06	19.91
*R*_free_*^b^* (%)	20.05	21.83
Number of atoms	44,629	44,738
Ramachandran plot		
Most favored region	5073 (95.61%)	5059 (95.34%)
Allowed region	233 (4.39%)	247 (4.66%)
Outliers	0 (0.00%)	0 (0.00%)

*^a^ R*_merge_ = Σ*_hkl_* |*I_hkl_* − 〈*I_hkl_*〉| /Σ*_hkl_ I_hkl_* where *I_hkl_* is the observed intensity and 〈*I*_hkl_〉 is the average intensity.

*^b^ R*_work_ = Σ*_hkl_* |*F_o_* − *F_c_*|*_hkl_*/Σ*_hkl_ F_ohkl_* for all data, except 5% which were used for *R*_free_ calculation.

The structure of MHCI_76_ pI matches very closely those of previously reported HLA-A2 MHCI structures; *e.g.* the root mean square deviation between MHCI_76_ and the MHCI structure used as model for the molecular replacement search (PDB code 1S9W) is 0.63 Å, calculated over all the C^α^ atoms of the assembled complex. WT MHCI pI and the MHCI_76_ pI complexes are also well superposable, displaying a root mean square deviation value of about 0.5 Å over the all C^α^ chains in the asymmetric unit. The D76N mutation site is localized in the β_2_m E-F loop, a region far from the heavy chain contact interface (Fig. 3*B*); accordingly, no major structural effects induced by the D76N mutation are observed at the heavy/light chain interface region. The β_2_m E-F loop within the MHCI_76_ structure superposes well on the corresponding region in the known WT MHCI structures and to the E-F loop of monomeric WT β_2_m (Fig. 3*C*). A 1.5 Å shift of Tyr^78^ toward Asn^76^, observed in the crystal structure of the monomeric D76N variant at 1.40 Å resolution (8), is not detected in the MHCI_76_ structure (Fig. 3*C*), perhaps as a result of the lower resolution of the MHCI_76_ crystals.

Overall, the crystal structure of MHCI_76_ pI does not appear to be affected by the D76N mutation; globally and locally, it matches closely the three-dimensional structures of WT MHCI pI. Furthermore, it is worth noting that residue 76 is not involved in any crystal contact with symmetry-related molecules within the crystal packing.

##### Protein Dynamics Assessment by Limited Proteolysis

**FIGURE 4.**
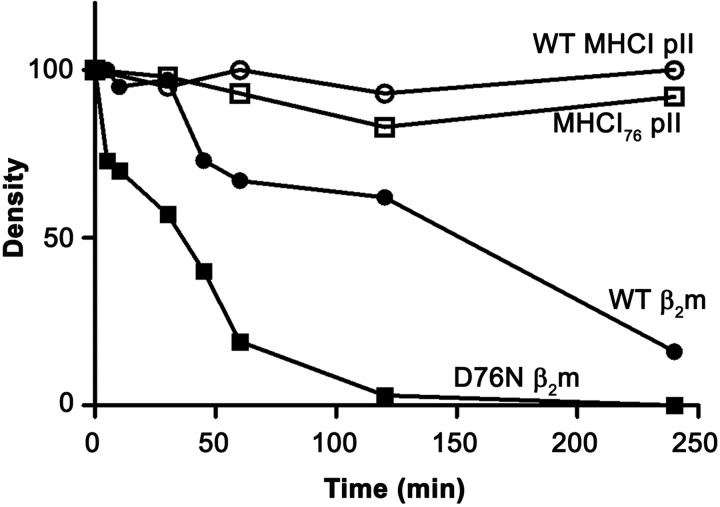
**Limited proteolysis of WT and variant β_2_m.** Kinetics of β_2_m tryptic degradation monitored by densitometric analysis of the corresponding SDS-PAGE bands are shown. A 1:200 trypsin:β_2_m ratio was used for proteolytic digestion of monomeric β_2_m isoforms. A 1:20 trypsin:MHCI ratio was used for the cleavage of the two β_2_m isoforms assembled within the MHCI.

**FIGURE 5.**
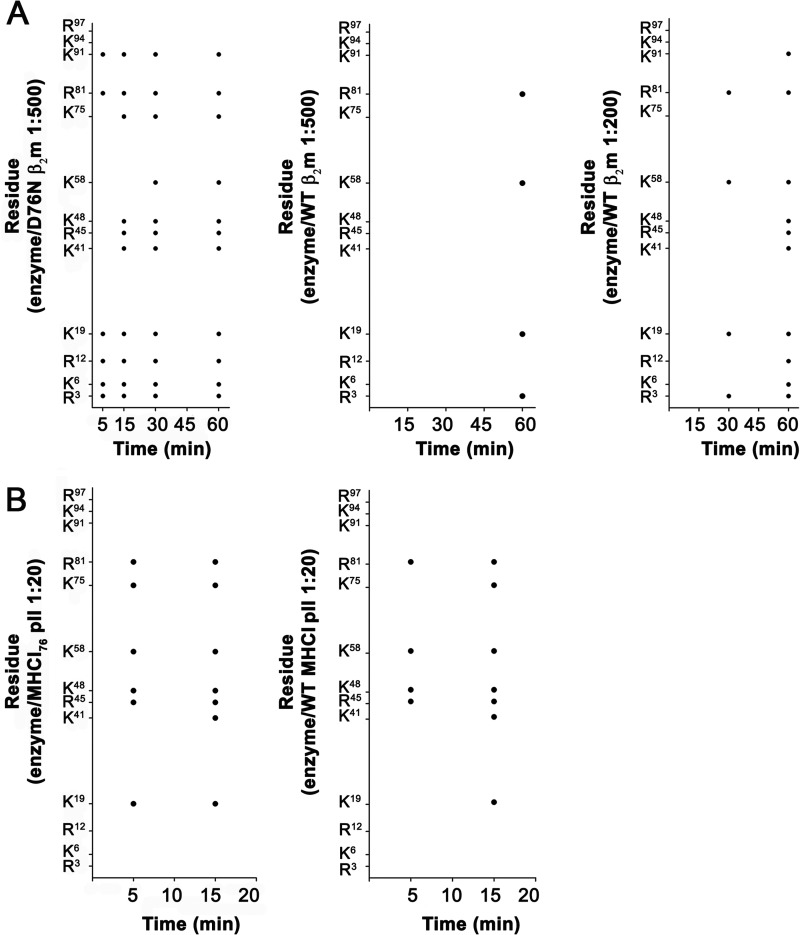
**Location of proteolytic sites.** *A*, trypsin cleavage sites in D76N and WT β_2_m for times shown and enzyme:protein ratios of 1:500 for the variant; 1/500 and 1/200 for WT β_2_m. *B*, trypsin cleavage sites in D76N variant and WT β_2_m within the MHCI complex.

A previous investigation of the conformational dynamics of the D76N β_2_m variant by NMR spectroscopy suggested that under native conditions, the main effect of the D76N mutation may lay in an overall perturbation of the dynamic properties of the protein (9). More precisely, an average attenuation of 5–10% of the backbone amide NOEs should correspond to a rigidity loss of the variant protein as compared with WT β_2_m that would extensively affect its intramolecular hydrogen-bonded network. To assess this hypothesis, monomeric WT and D76N variant β_2_m were analyzed by limited proteolysis. This approach has been successfully used, by us and others, to assess the flexibility and dynamics of several proteins, including WT β_2_m and its ΔN6 truncated form (30–32). Thus, limited proteolysis experiments were carried out on WT and D76N β_2_m molecules, both in their free forms and assembled within MHCI complexes. Two different series of experiments were performed. Firstly, the overall susceptibility to proteolytic digestion of the variant as compared with WT β_2_m was monitored (Fig. 4); secondly, the specific sites sensitive to proteolytic cleavages were identified (Fig. 5).

Fig. 4 clearly shows that monomeric D76N β_2_m is more rapidly degraded than the WT protein when exposed to trypsin, at an enzyme/β_2_m ratio of 1/200. When the same experiment was carried out on the two β_2_m species associated in their MHCI assembly, at a trypsin/MHCI ratio of 1/200, both β_2_m species turned out to be fully protected from proteolytic digestion. A high degree of protection can also be observed for WT β_2_m. Significant digestion was only obtained by increasing the concentration of protease to a 1/20 ratio, and specific cleavages are reported in Fig. 5.

The high cleavage specificity of trypsin allows identification of the main sites of proteolysis and comparison of the digestion pattern of the two β_2_m species both in their monomeric forms and in their assembled MHCI. In particular, comparison of the early cleavage sites can provide information on the conformational dynamics of the two proteins, whereas the late cleavages may derive from further proteolysis of primary digestion peptides and will not be discussed. Even at very low enzyme/β_2_m ratios (1/500) and very short incubation times (5 min), specific cleavages are detectable for the D76N variant. In particular, D76N β_2_m is rapidly cleaved at residues Arg^3^, Lys^6^, Arg^12^, Lys^19^, Arg^81^, and Lys^91^. At the same enzyme/β_2_m ratio, cleavages become detectable in the WT protein only after 60 min of incubation, at residues Arg^3^, Lys^19^, Lys^58^, and Arg^81^ (Fig. 5*A*). The same preferential sites of cleavage are confirmed using shorter incubation times (30 min) at higher trypsin concentration (1/200) (Fig. 5*A*).

Limited proteolysis therefore highlights residues Lys^6^, Arg^12^, and Lys^91^ as cleavage sites specific to the D76N variant. Cleavage at residue Lys^6^ proves consistent with the NMR evidence for the long distance destabilizing effect on the β_2_m A strand, linked to the D76N mutation (9). It is worth mentioning that trypsin cleavage at residue 6 is a primary event in the controlled proteolysis of WT β_2_m fibrils, whereas the same cleavage does not occur in native WT β_2_m in solution (32, 33); most importantly, destabilization of β_2_m A strand is pivotal for β_2_m amyloid aggregation (31). Thus, considering the cleavage at residue Lys^6^ as a sign of increased structural dynamics, the proteolytic event is consistent with the high aggregation propensity of the D76N variant. The cleavage at residue Arg^12^, located at the edge of the A strand, confirms the increased flexibility of the A strand linked to the D76N mutation. Conversely, the early cleavage at Lys^91^, occurring rapidly in D76N variant β_2_m, indicates a destabilization of the G strand; residue Lys^91^ plays a role in structuring the G strand with hydrogen bonds to residues 82, 83, and 89. Conservation of this H-bonded network is essential for the stability of the G strand.

The investigation of specific cleavage sites on both β_2_m variants, assembled in their respective MHCI, confirms a remarkable protective effect of the heavy chain on the two β_2_m species. The digestion patterns of WT MHCI and MHCI_76_, at 1/20 enzyme/protein ratios, are very similar in terms of cleavage specificity and kinetics of digestion. Interestingly, the three early proteolysis sites observed in free D76N (Lys^6^, Arg^12^, Lys^91^) remain uncleaved following association with the heavy chain. In this respect, whereas Arg^12^ is indeed buried in the assembled MHCI (hence protected from protease access), Lys^6^ and Lys^91^ are both solvent-accessible residues in the assembled MHCI. Therefore, the absence of proteolytic cleavage at these two sites might derive from a decrease in β_2_m conformational fluctuations and from an overall stabilization of the hydrogen-bonded networks in these solvent-accessible protein regions.

##### Expression and Localization of the D76N Variant in Vivo

**FIGURE 6.**
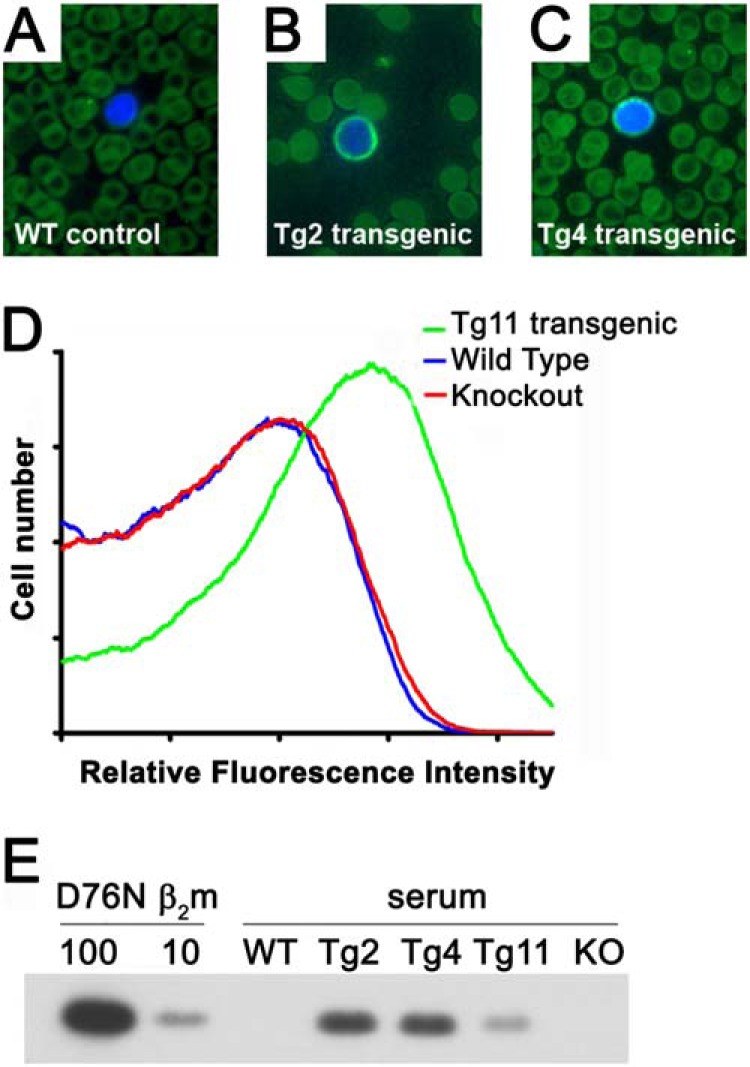
**Human β_2_m expression in human D76N β_2_m transgenic mice.** D76N β_2_m is expressed by three independent transgenic lines of mice (Tg2, Tg4, and Tg11), all on the wild type C57BL/6J background. *A–C*, direct immunofluorescence of blood films stained with FITC-labeled anti-human β_2_m monoclonal antibody B2M-01. In each panel, a single representative leukocyte is shown, identified by counterstaining with the blue fluorescent DNA stain Hoechst 33342; note that most cells visible in each field are erythrocytes, which are intrinsically autofluorescent, lack β_2_m on the surface, and do not possess nuclei. *A*, negative control nontransgenic C57BL/6J mouse showing minimal signal; *B* and *C*, leukocytes of transgenic mice of lines Tg2 and Tg4 show a bright halo of D76N β_2_m staining. *D*, FACS analysis of live peripheral blood leukocytes demonstrating cell surface expression of D76N β_2_m (*green line*, Tg11 mouse). The FACS profiles of control nontransgenic littermate (*blue*) and mouse β_2_m knockouts (*red*) are indistinguishable, confirming that the anti-human β_2_m monoclonal antibody lacks cross-reactivity with endogenous mouse β_2_m. *E*, Western blot analysis shows expression of free human β_2_m in the blood of D76N β_2_m transgenic mice (lines Tg2, Tg4, and Tg11) as a 12-kDa band co-migrating with pure recombinant D76N β_2_m (100 or 10 ng loaded, as indicated). No signal was detected in wild type C57Bl/6J (*WT*) or in knock-out mice (*KO*) that do not express endogenous β_2_m; 1 μl of serum was loaded in each case.

We have created transgenic mice expressing D76N variant β_2_m to provide a mouse model of systemic β_2_m amyloidosis. The characterization of this model will be reported elsewhere, but here we show the cell surface expression of D76N variant β_2_m in the MHCI complex. Although the transgenic mice do not express human class I heavy chains, it is well established that normal human β_2_m associates with mouse class I heavy chains (34, 35). Indeed leukocytes in blood films of transgenic mice were specifically stained following incubation with a directly labeled monoclonal antibody specific for human β_2_m, whereas those of nontransgenic mice were not (Fig. 6, *A–C*). This result is consistent with cell surface localization of human D76N variant β_2_m but, although the cells were not permeabilized, it is conceivable that the signal reflects the β_2_m protein within the cells. Cell surface localization of D76N β_2_m was therefore also assessed in living cells by FACS analysis following incubation with FITC-labeled monoclonal anti-human β_2_m antibody. Cells of normal and β_2_m knock-out mice gave indistinguishable patterns of background fluorescence, whereas D76N β_2_m transgenic mouse cells had significantly higher signal (Fig. 6*D*), showing that human D76N β_2_m is present on the cell surface. Detection of transgenic D76N β_2_m in the serum of transgenic mice by ELISA (not shown) and Western blotting (Fig. 6*E*) at levels comparable with or higher than those normally observed in humans provides further evidence that D76N β_2_m escapes cellular quality control mechanisms, and is consistent with the *in vitro* observation, which shows that the assembly of β_2_m within the complex masks its intrinsic misfolding propensity.

**FIGURE 7.**
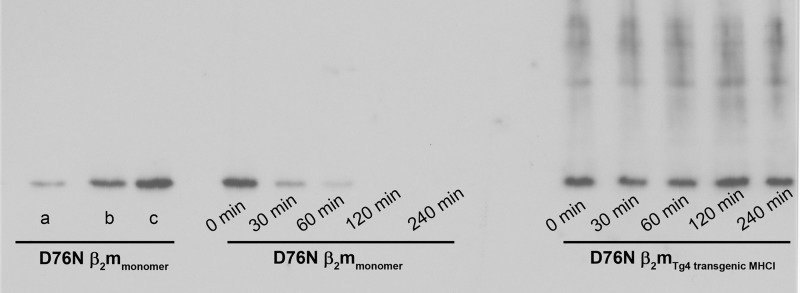
**D76N β_2_m transgenic leukocytes exposed to trypsin.** Shown is a Western blot of D76N β_2_m (4 μg/ml) in both monomeric and transgenic mouse leukocyte-associated forms at time 0, 30, 60, 120, and 240 min after trypsin digestion at a 1:20 enzyme:substrate ratio. Controls of recombinant human variant β_2_m are also included (*a*, 1.25 μg/ml; *b*, 2.5 μg/ml; *c*, 5 μg/ml). The leukocytes were obtained from line 4 transgenic mice.

To confirm whether the natural form of D76N β_2_m is protected from the proteolytic cleavage when assembled in the MHCI complex in its physiological environment, we have exposed mononucleated cells, extracted from two spleens of our transgenic mice, to trypsin, and we have monitored the digestion of the β_2_m variant. The results reported in Fig. 7 indicate that the protein is highly protected from digestion when physiologically assembled in a membrane-anchored MHCI. Our data confirm the results obtained for the soluble recombinant MHCI complex and suggest that the cell membrane might contribute to stabilize the protein and protect it from proteolysis. Indeed it has been predicted that a significant surface of β_2_m is in contact with the membrane and therefore not even accessible to monoclonal antibodies recognizing epitopes in the β_2_m C-terminal region (36).

## DISCUSSION

Although human cells adopt sophisticated and potent systems to control and minimize protein misfolding (2), a limited number of proteins manage to escape the cellular quality control and aggregate into amyloids. Proteins responsible for amyloid-related disorders either show their amyloidogenic propensity after passing the cell quality test or are stable proteins that begin to aggregate only under specific pathophysiological conditions (*i.e.* an abnormally elevated plasma concentration) (37). The D76N variant β_2_m appears to be an odd case in this context. On the one hand, this β_2_m variant is extremely prone to misfold and self-aggregate under physiological conditions, even at very low concentrations (8). On the other hand, in patients, the D76N variant is neither efficiently recognized nor destroyed by the UPR system, having been identified in massive extracellular amyloid deposits (8).

Our data explain these apparently contrasting observations. Once β_2_m is synthesized in the cell, it is promptly assembled together with a heavy chain and a peptide to form the MHCI, which is then transported to the cell surface (7). In this way, the amyloidogenic D76N β_2_m variant has only a transient existence intracellularly as an isolated chain. Our data show that the association within the MHCI has a remarkable stabilizing effect on the D76N β_2_m variant.

Despite the much reduced stability of the isolated D76N variant as compared with WT β_2_m, its structure, stability, and dynamics closely match those of WT β_2_m once assembled within the MHCI. In particular, the results of limited proteolysis indicate that the interaction with the heavy chain induces an overall stabilization of the variant β_2_m, which extends beyond the heavy chain association interface. Therefore, the assembly of the MHCI would act as a chaperone that stabilizes the variant and protects the cell from D76N β_2_m aggregation. However, in fulfilling such a role, the MHCI_76_ molecules mask and hide the misfolding propensity of the D76N variant that escapes from the UPR and other cellular quality control pathways. Hence, although MHCI_76_ is crucial for the transfer of this life-threatening β_2_m variant out of the cell, it acts as a Trojan horse for translocation of the D76N β_2_m variant to the membrane. Once exposed on the membrane surface, the MHCI_76_ undergoes the physiological shedding of β_2_m, and the circulating free D76N β_2_m chains can ultimately disclose their misfolding and fibrillogenic propensity.

The elucidation of the molecular effects of MHCI assembly on the stability and dynamics of the β_2_m D76N variant highlights the potential for the therapeutic application of β_2_m interactors, including small molecules (38) or nanobodies (39), which can stabilize the variant and protect against the amyloid transition. It is known that 17b-estradiol decreases MHCI production (40), and to do this, clinically acceptable treatments may provide an additional avenue to control D76N β_2_m-dependent systemic amyloidosis. The availability of the transgenic mice expressing the variant will represent a valuable tool for testing this hypothesis.

## Footnotes

4 The abbreviations used are:

β_2_m: β_2_-microglobulin
WT β_2_m: wild type β_2_-microglobulin
MHCI: class I major histocompatibility complex
MHCI_76_: class I major histocompatibility complex containing D76N β_2_-microglobulin
WT MHCI: class I major histocompatibility complex containing wild type β_2_-microglobulin
UPR: unfolded protein response
nano-ESI-MS: nano electrospray ionization mass spectrometry
WT MHCI pI: complex containing heavy chain HLA-A2 with WT β_2_m and peptide NY-ESO-1(Y^1^V^9^)
MHCI_76_ pI: complex containing heavy chain HLA-A2 with D76N β_2_m and peptide NY-ESO-1(Y^1^V^9^)
WT MHCI pII: complex containing heavy chain HLA-A2 with WT β_2_m and peptide FR-20(Y^3^)
MHCI_76_ pII: complex containing heavy chain HLA-A2 with D76N β_2_m and peptide FR-20(Y^3^)
DP: declustering potential
Fmoc: *N*-(9-fluorenyl)methoxycarbonyl.
